# The gut–lung axis in asthma: microbiota-driven mechanisms and therapeutic perspectives

**DOI:** 10.3389/fmicb.2025.1680521

**Published:** 2025-10-28

**Authors:** Zihan Yang, Wei Mao, Junyang Wang, Leyi Yin

**Affiliations:** Huzhou Traditional Chinese Medicine Hospital Affiliated to Zhejiang Chinese Medical University (Huzhou Hospital of Traditional Chinese Medicine), Huzhou, China

**Keywords:** gut microbiota, asthma, gut-lung axis, immunomodulation, probiotics

## Abstract

Asthma is a multifactorial inflammatory airway disease shaped by complex interactions among host genetics, environmental exposures, and the microbiota. The human body hosts a highly diverse microbial ecosystem, comprising more than 10,000 species that profoundly influence host physiology through the regulation of nutrient metabolism and immune homeostasis. Disruption of this balance, or dysbiosis, contributes to the onset and progression of immune-mediated diseases, including asthma. Asthma is a multifactorial disorder driven by the complex interaction of genetic susceptibility and environmental exposures, and its heterogeneous phenotypes and severity are increasingly associated with alterations in the microbiota. In particular, the gut–lung axis represents a critical bidirectional pathway through which microbial communities and their metabolites in the gut and airways shape immune responses and respiratory health. This review summarizes current evidence on microbiota-driven mechanisms underlying asthma pathogenesis, highlights the role of the gut–lung axis in immune regulation, and discusses emerging microbiota-targeted therapeutic strategies, emphasizing their potential for clinical translation in asthma treatment.

## 1 Introduction

Asthma is a chronic and heterogeneous respiratory disorder characterized by persistent airway inflammation and variable expiratory airflow obstruction (Global Initiative for Asthma, 2025). Clinically, it manifests as recurrent wheezing, dyspnea, chest tightness, and cough, affecting more than 300 million people globally and imposing a substantial socioeconomic burden on healthcare systems and society (Wang et al., 2023). The pathogenesis of asthma involves multiple interrelated processes, including airway hyperresponsiveness (AHR), structural remodeling, T helper (Th) cell imbalance, eosinophilic infiltration, and goblet cell hyperplasia (Maia et al., 2024). Together, these alterations underlie the chronic inflammatory state and phenotypic variability observed in patients.

In recent years, increasing attention has focused on the role of the microbiota as a critical modulator of immune development and respiratory health. The human gastrointestinal tract harbors a dense and diverse microbial community that contributes to nutrient metabolism, epithelial barrier integrity, and the regulation of local and systemic immune responses (Kandari et al., 2024). Although the microbial biomass of the lung is relatively low, its community structure is shaped by inhalation, mucosal dispersion, and host–microbe interactions (Druszczynska et al., 2024). Communication between the gut and lung occurs through lymphatic circulation, hematogenous dissemination, and micro-aspiration, forming a bidirectional regulatory network known as the gut–lung axis. In this review, we define the gut–lung axis as the bidirectional communication between intestinal and respiratory systems mediated by microbial communities, metabolites, and immune signaling pathways. Dysbiosis refers to a state of microbial imbalance characterized by reduced diversity and altered functional capacity, whereas immune regulation encompasses microbial modulation of both innate and adaptive immune responses.

Accumulating evidence indicates that alterations in gut microbial composition are associated with asthma susceptibility, phenotypic expression, and disease progression. Characteristic changes include reduced abundance of beneficial commensals such as *Bifidobacterium* and *Bacteroides fragilis*, as well as expansion of potentially pro-inflammatory taxa (Enaud et al., 2020). These microbial imbalances disrupt immune homeostasis, enhance Th2 and Th17 polarization, and promote airway hyperresponsiveness and inflammation. Furthermore, gut dysbiosis has been linked to broader systemic effects through the gut–lung axis, shaping pulmonary immune responses via metabolite signaling and immune regulation. Such disturbances are increasingly recognized as key contributors to asthma heterogeneity, influencing not only disease mechanisms but also clinical phenotypes and therapeutic outcomes.

To improve transparency and reduce citation bias, we searched PubMed and Web of Science for relevant studies published between 2000 and 2025 using combinations of the keywords “asthma,” “gut–lung axis,” “microbiota,” and “immune regulation.” Additional articles were identified through manual screening of reference lists.

Unlike previous reviews that have primarily focused on individual mechanisms or clinical associations, this article proposes a hierarchical framework that classifies microbiota–host interactions into primary drivers, intermediate modulators, and downstream immune effectors. This structure integrates recent advances in metabolite signaling, epigenetic regulation, and neuro–immune crosstalk, providing a more unified mechanistic perspective on the gut–lung axis in asthma. In this review, we synthesize current evidence on microbiota–immune interactions in asthma, focusing on the gut–lung axis as an integrative framework. We further discuss recent progress in microbiota-targeted therapeutic strategies and highlight remaining knowledge gaps to inform future mechanistic and clinical research.

## 2 Microorganisms and asthma pathogenesis

**Figure 1:**
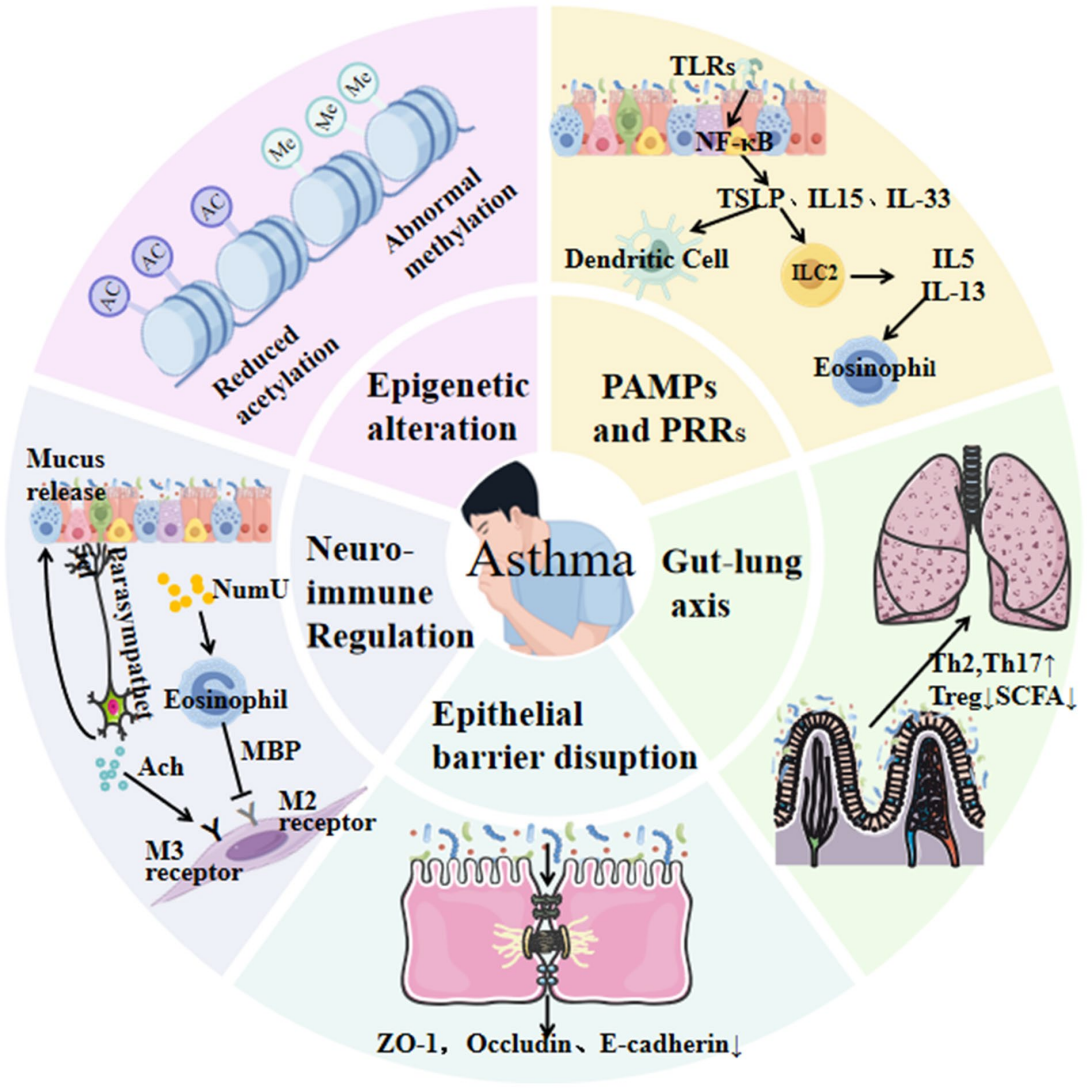
This schematic illustrates key pathways linking microbiota to asthma, including innate immune activation (e.g., TLRs/NF-κB), epithelial barrier disruption, gut–lung axis interactions via reduced short-chain fatty acid (SCFA) production, Th2/Th17 polarization, and neuro-immune crosstalk.

The development and progression of asthma are shaped by intricate host–microbe interactions, which influence immune maturation, epithelial barrier integrity, and inflammatory responses. Early conceptual frameworks, such as the hygiene hypothesis, proposed that reduced exposure to pathogens during early childhood impairs immune tolerance and increases the risk of allergic diseases. This idea has since evolved into the biodiversity hypothesis, which emphasizes that limited contact with diverse natural environments and environmental microbiota diminishes immune regulatory capacity and heightens susceptibility to asthma and other allergic disorders (Haahtela et al., 2013). Supporting this view, decreased diversity of commensal taxa—including *Lactobacillus*, *Bifidobacterium*, and *Bacteroides*—has been associated with impaired induction of regulatory T (Treg) cells and disrupted immune homeostasis, ultimately enhancing vulnerability to airway inflammation. Current evidence indicates that the influence of the microbiota on asthma involves multiple, interconnected mechanisms that operate across different biological levels. These mechanisms encompass innate immune sensing through pattern recognition receptors (PRRs), modulation of epithelial barrier structure and function, epigenetic regulation, and neuro–immune signaling. Collectively, they can be broadly classified into primary drivers, intermediate modulators, and downstream immune effectors, reflecting the hierarchical organization of microbiota–host interactions in asthma. Figure 1 provides an integrative schematic of these pathways, illustrating how gut microbial communities act as upstream drivers, epithelial and epigenetic processes function as intermediate modulators, and neuro–immune interactions operate as downstream regulatory mechanisms. This framework contextualizes the subsequent sections within a unified model of asthma pathogenesis.

### 2.1 Activation of pathogen-associated molecular patterns and pattern recognition receptors

Airway epithelial cells serve as the first line of defense by recognizing microbial threats through pattern recognition receptors (PRRs) such as Toll-like receptors (TLRs). Upon detection of pathogen-associated molecular patterns (PAMPs) and damage-associated molecular patterns (DAMPs), these cells initiate downstream signaling cascades—most notably NF-κB—which drive the production of cytokines and chemokines that shape both innate and adaptive immune responses in asthma (Komlósi et al., 2022). PRR activation exhibits marked pathogen specificity. In the gut, bacterial components are primarily sensed through TLR2, TLR4 (lipopolysaccharide, LPS), and NOD1/NOD2 (peptidoglycan), whereas viral signals are detected by TLR3, TLR7/8, RIG-I, and MDA-5, which recognize viral RNA. Although the pulmonary microbiome contributes to PRR-mediated signaling, its impact is generally secondary to that of gut-derived stimuli, reflecting the higher microbial biomass and metabolic activity of the gastrointestinal tract (Komlósi et al., 2022). Importantly, the consequences of PRR engagement in asthma are highly context dependent. Zakeri et al. demonstrated that the effects of TLR agonists vary according to cell type (hematopoietic vs. non-hematopoietic), allergen characteristics (e.g., house dust mite (HDM), ovalbumin), and the route of administration, collectively determining whether PRR signaling dampens, exacerbates, or promotes disease development. Headland et al. further showed that gut microbiota–derived LPS induces elevated oncostatin M (OSM) expression through the TLR4–MyD88 pathway, driving airway inflammation and mucus hypersecretion characteristic of severe asthma. Consistently, OSM levels are markedly increased in the airways of patients with non–type 2 severe asthma (Headland et al., 2022). Moreover, Porsbjerg and colleagues reported that bronchial epithelial responses to viral mimetics differ across asthma phenotypes and severities, highlighting the heterogeneity of PRR-mediated pathways (Porsbjerg et al., 2022). Collectively, these studies indicate that PRR signaling exerts bidirectional immunomodulatory effects in asthma: gut microbial signals act as central upstream regulators, whereas their downstream consequences are shaped by microbial type, host genetic background, and the local immune microenvironment.

### 2.2 Epithelial barrier disruption and aberrant repair mechanisms

Under physiological conditions, epithelial cells in both the gut and respiratory tract maintain barrier integrity by producing immunoglobulin A (IgA), defensins, and lysozyme, thereby preventing microbial invasion and regulating immune homeostasis (Losol et al., 2023). Repeated allergen exposure in individuals with asthma leads to structural and functional barrier disruption, which profoundly alters local immune responses. Upon injury, epithelial cells release alarmins such as interleukin (IL) -25, IL-33, and thymic stromal lymphopoietin (TSLP), which activate group 2 innate lymphoid cells (ILC2s) and initiate type 2 inflammatory cascades (Losol et al., 2023). In parallel, epithelial cells interact with dendritic cells (DCs) and other immune cells, amplifying cytokine secretion and promoting airway inflammation.

A variety of microbial pathogens exploit these vulnerabilities to disrupt epithelial homeostasis. For example, the fungal allergen alkaline protease 1 from *Aspergillus* directly damages bronchiolar cell–cell junctions in murine models by inducing calcium influx via the calcineurin pathway, thereby promoting Th cell–mediated pulmonary eosinophilia (Wiesner et al., 2020). Similarly, respiratory viral infections exert potent barrier-modulating effects. Respiratory syncytial virus (RSV) downregulates tight junction proteins zonula occludens-1 (ZO-1) and E-cadherin through the IL-33/ST2/MyD88 pathway (Miao et al., 2025), while human rhinovirus (HRV) remodels epithelial junctions through IL-15–dependent antiviral responses, leading to enhanced permeability and exacerbated Th2 inflammation (Looi et al., 2016). These microbial insults act synergistically with impaired epithelial repair mechanisms and heightened immune activation to produce sustained barrier dysfunction in both the gut and lungs. Importantly, accumulating evidence indicates that gut dysbiosis serves as a critical upstream driver of these processes, linking microbial community alterations to airway inflammation through barrier disruption and alarmin release. Collectively, epithelial barrier dysfunction represents a key intermediate mechanism connecting gut microbial disturbances to asthma pathogenesis.

### 2.3 Microbiota-host interactions

The gut and lung are connected through the gut–lung axis, a bidirectional communication network shaped by their shared embryological origin and mucosal immune system. In healthy individuals, the gut microbiota is dominated by the phyla *Firmicutes*, *Bacteroidetes*, and *Actinobacteria*, along with less abundant taxa such as *Fusobacteria*. This microbial community plays a pivotal role in immune education, metabolic regulation, and epithelial barrier maintenance. Among gut-derived metabolites, SCFAs are key mediators of protective effects. Reduced fecal SCFA concentrations in pediatric atopic disorders support their role in maintaining immune tolerance and suppressing inflammation (Sun et al., 2017; Kumari and Kozyrskyj, 2017). SCFAs modulate epithelial cytokine production—including IL-6 and IL-8—through TLR and NF-κB signaling pathways, thereby exerting broad anti-inflammatory functions. Specifically, butyrate alleviates asthma by suppressing GATA3 expression and reducing IL-5 and IL-13 secretion from ILC2s (Lewis et al., 2019), whereas propionate acts through free fatty acid receptor 3 to promote DC and macrophage differentiation, attenuating allergic inflammation (Zhou et al., 2021). Maternal microbial exposure also influences offspring immunity through epigenetic and metabolic pathways. Perdijk et al. demonstrated that maternal high-fiber or acetate-enriched diets protect offspring against allergic airway disease by inhibiting histone deacetylase 9 (HDAC9) and promoting Foxp3^+^ Treg cell differentiation (Perdijk et al., 2024). Beyond SCFAs, other metabolite classes such as bile acids and polyamines also modulate airway inflammation. For instance, tauroursodeoxycholic acid (TUDCA) significantly reduces HDM-induced IL-4, IL-5, IL-13, and IgE levels, whereas β-muricholic acid suppresses IL-6 and IL-33 production, underscoring the diverse metabolic pathways through which gut microbes influence asthma pathogenesis (Nakada et al., 2019).

Although recent research has focused on the gut microbiota, the respiratory microbiome also contributes to disease heterogeneity. In healthy adults, the lower airways are typically dominated by *Streptococcus*, *Veillonella*, and *Prevotella*. Phenotype-specific shifts have been observed in asthma: eosinophilic asthma is associated with enrichment of Actinobacteria (Simpson et al., 2016), whereas neutrophilic asthma is characterized by expansion of Proteobacteria, particularly *Haemophilus influenzae*. Versi et al. showed that *H. influenzae* dominance correlates with neutrophilic inflammation, prolonged disease duration, frequent exacerbations, high corticosteroid use, and elevated serum IL-8, while fractional exhaled nitric oxide (FeNO) and eosinophilic inflammation are increased only in a subset of patients (Versi et al., 2023). These observations indicate that *H. influenzae* enrichment reflects neutrophil-driven, rather than T2-high eosinophilic, inflammation.

Regional and longitudinal studies further highlight the systemic impact of gut microbes on asthma phenotypes. Cross-sectional research in South China revealed distinct gut microbial signatures across asthma endotypes, linking altered abundances of *Bacteroides*, *Bifidobacterium*, and SCFA-producing taxa with specific clinical manifestations (Zou et al., 2021). Similarly, data from a Chinese birth cohort showed that early-life dynamics of *Bifidobacterium*, *Faecalibacterium*, and fecal SCFA levels were associated with subsequent development of eczema and wheezing (Cheung et al., 2023). A recent systematic review confirmed that children with asthma or recurrent wheeze consistently exhibit reduced fecal SCFA concentrations (Sasaki et al., 2024). Collectively, these findings highlight the central role of gut microbial diversity and metabolite production in shaping airway immune responses, supporting the gut–lung axis as a key determinant of asthma pathogenesis.

Finally, certain commensal respiratory taxa confer protective effects. Genera such as *Corynebacterium* and *Dolosigranulum* can inhibit pathogenic *Streptococcus* colonization by producing antimicrobial compounds, thereby lowering the risk of poorly controlled asthma in children (Zhou et al., 2019). These protective interactions emphasize that the microbiome—particularly gut microbial metabolites—modulates asthma pathophysiology through systemic immune regulation and inter-organ communication, while respiratory communities add an additional layer of modulation that contributes to disease heterogeneity.

### 2.4 Epigenetic modifications

Epigenetic mechanisms regulate gene expression without altering the underlying DNA sequence and include DNA methylation, histone modifications, and non-coding RNA regulation (Wu et al., 2021). These modifications shape immune development and airway inflammation by modulating the accessibility of key regulatory loci. Host epigenetic programs are highly sensitive to microbial and metabolic signals, positioning the microbiota as an upstream driver of epigenetic remodeling in asthma.

Microbial metabolites influence host epigenetics through several complementary pathways. One of the best-characterized examples is butyrate, a major SCFA produced by commensal gut bacteria. Schulthess et al. demonstrated that butyrate promotes monocyte differentiation into macrophages by inhibiting histone deacetylase 3 (HDAC3), thereby reducing mammalian target of rapamycin kinase activity, enhancing LC3-mediated antimicrobial responses, and inducing antimicrobial peptide production (Schulthess et al., 2019). These epigenetic changes augment host defense and modulate immune activation, providing a mechanistic link between microbial metabolites and asthma susceptibility.

Microbiota-mediated epigenetic reprogramming can also occur through community-level alterations. Ramar et al. showed that gut microbiota transplantation (GMT) from asthma-susceptible donors induced dysregulated butyrate metabolism in recipient mice, leading to altered DNA methylation patterns in DCs (Ramar et al., 2025). This reprogramming enhanced antigen-presenting capacity and T cell proliferation, epigenetically amplifying allergic inflammation. These findings highlight epigenetic modification as a mid-level mechanism that integrates upstream microbial signals with downstream immune responses.

Epigenetic programming during early life represents another critical determinant of asthma susceptibility. DeVries and colleagues reported that maternal IFN-γ to IL-13 ratios shape DNA methylation profiles in cord blood mononuclear cells (CBMCs), altering innate immune training and nasal microbiota succession in offspring (DeVries et al., 2022). Maternal diet, microbial exposure, and metabolite availability thereby exert long-term influences on immune trajectories through epigenetic pathways, potentially predisposing individuals to asthma.

Epigenetic regulation also modulates therapeutic responses. Clinical research has shown that inhaled corticosteroids influence epigenetic regulators—including IL-2, TNF-α, NF-κB, and CCAAT/enhancer-binding protein pathways—in parallel with microbiome remodeling (Perez-Garcia et al., 2025). These results suggest that epigenetic states not only reflect disease susceptibility but also determine treatment efficacy, providing a mechanistic rationale for combining microbiota-targeted interventions with epigenetic modulation in future precision therapies.

Collectively, these studies position epigenetic modifications as a critical intermediate layer linking microbial signals, immune programming, and clinical outcomes in asthma. Future research should prioritize clarifying the causal directionality of these interactions, identifying key microbial–epigenetic–immune nodes, and standardizing longitudinal epigenomic profiling strategies to facilitate clinical translation.

### 2.5 Neuro-immune regulation

The pathogenesis of asthma involves dynamic interactions among the nervous system, immune system, and the microbiota. Neurotransmitters and neuropeptides released by nerve cells rapidly activate immune effector pathways, inducing the production of inflammatory mediators while simultaneously shaping local microbial communities (Joos et al., 2003). Conversely, immune cell–derived mediators such as histamine and neurotrophic factors stimulate sensory neurons, promoting bronchoconstriction and cough reflexes. This bidirectional neuro–immune communication contributes to asthma pathology by regulating type 2 inflammatory processes (Voisin et al., 2017). Recent studies have highlighted the role of the gut–brain–lung axis as a key pathway linking microbial signals to neural and immune responses. In murine models, postprandial acetylcholine release by the parasympathetic nervous system activates ILC2s through muscarinic receptor M4 (Chrm4), leading to increased secretion of IL-5 and IL-13 and exacerbated airway inflammation (Chen et al., 2025). Conversely, genetic deletion of Chrm4 significantly attenuates ILC2-driven airway pathology. These findings indicate that gut microbiota–derived metabolites, particularly butyrate, can modulate neural circuits via the dorsal vagal nucleus, thereby amplifying Th2 cytokine responses and downstream inflammatory cascades.

Under conditions of persistent Th2 inflammation, neutrophils release chromatin and granule proteins to form neutrophil extracellular traps (NETs). While NETs facilitate antifungal defense—for example, against *Candida albicans*—they also promote dysbiosis and sustain chronic inflammation (Daniel et al., 2019). This reciprocal interaction between neural pathways, immune responses, and microbial communities contributes to disease persistence and heterogeneity in asthma.

Collectively, microorganisms across different kingdoms—including bacteria, viruses, and fungi—shape asthma pathogenesis through multiple mechanisms, including disruption of epithelial barriers, modulation of innate and adaptive immunity, epigenetic remodeling, and neuro–immune signaling. These neuro–immune interactions represent a downstream regulatory layer that integrates microbial and immune signals, contributing to both acute responses and chronic disease trajectories. Table 1 provides an overview of key microorganisms and their mechanistic contributions to asthma pathogenesis based on experimental and clinical studies.

**Table 1:** Mechanisms of airway and gut microbes in asthma pathogenesis.

Microbiota/agent	Study model	Key molecules/pathways	Effect on asthma pathogenesis
Bacteria			
*Klebsiella pneumoniae* or LPS	Murine model	LPS/TLR4/MyD88/OSM	OSM drives severe asthma pathophysiological features (Headland et al., 2022)
*Bifidobacterium* spp.	Murine model	12,13-diHOME, PPARγ	Reduces anti-inflammatory cytokine secretion and Treg cell numbers (Levan et al., 2019)
*Pseudomonas aeruginosa*	Murine model	RIPK1-RIPK3-MLKL	Causes goblet cell metaplasia, mucus hypersecretion, epithelial damage, and steroid resistance (Guan et al., 2024)
Viruses			
Respiratory syncytial virus (RSV)	Murine model	IL-33/ST2/MyD88	Disrupts airway epithelial barrier (e.g., ZO-1, Occludin, E-cadherin) (Miao et al., 2025)
Human rhinovirus 1B (HRV-1B)	Murine model	IRF3/IFN-α/IFN-β, IL-15	Alters tight junction expression, increases epithelial permeability (Looi et al., 2016)
Enterovirus A71 (EV-A71)	Murine model	Bone marrow-derived macrophage-sustained innate immune memory	Exacerbates allergen-induced airway inflammation following neonatal infection (Chen et al., 2021)
Poly(I: C)	Murine model	YAP/FOXM1/SPDEF/MUC5AC	Promotes goblet cell hyperplasia, mucus hypersecretion, AHR, and lung inflammation (Bu et al., 2023)
Fungi			
*Candida parapsilosis*	Murine model	PGE₂	Gut fungal overgrowth promotes distant M2 macrophage activation, influencing allergic inflammation (Kim et al., 2014)
*Aspergillus amstelodami*, etc.	Murine model	Syk signaling	Fungal sensing by gut CX3CR1 + MNPs aggravates allergic airway disease (Li et al., 2018)
*Aspergillus fumigatus*	Murine model	ECM, Ca^2+^	Alp1-mediated ECM degradation evokes RhoA-dependent Ca^2+^ sensitivity and bronchoconstriction (Li et al., 2018)
*Aspergillus fumigatus*	Murine model	TRPV4, calcium, calcineurin	Alp1 damages bronchiolar cell junctions, triggering calcineurin-mediated inflammation in club cells (Wiesner et al., 2020)
*Alternaria alternata*	Murine model	Ceramide	Ceramide elevation contributes to apoptosis, ROS generation, and neutrophilic inflammation (James et al., 2021)
*Alternaria alternata* (Alt a 1)	Murine model	Alt a 1, TLR2/4, MYD88, TIRAP	Alt a 1 induces potent cytokine/chemokine responses and innate immunostimulatory activities in bronchial epithelial cells
*Wallemia mellicola*	Murine model	Unknown	Enhances severity of allergic airways disease (Skalski et al., 2018)
Other			
*Marvinbryantia*, *Clostridium*	Murine model	Succinate/SUCNR1/IL-1β	Exacerbates allergic airway inflammation by promoting protein succinylation (Wang et al., 2025)

AHR, Airway hyperresponsiveness; ECM, Extracellular matrix; MNPs, Mononuclear phagocytes; OSM, Oncostatin M; PGE₂, Prostaglandin E_2_; Treg, Regulatory T cells.

## 3 The clinical correlation between microorganisms and asthma

### 3.1 Microbial diversity and asthma phenotypes

Asthma exhibits marked clinical heterogeneity, posing significant challenges for disease management. Based on predominant inflammatory cells identified in induced sputum or bronchial biopsy—such as eosinophils, neutrophils, and lymphocytes—patients are commonly categorized into distinct inflammatory phenotypes. Among these, the Th2-high phenotype (typically associated with eosinophilic inflammation) and the Th2-low or non-Th2 phenotype (often linked to neutrophilic or lymphocytic inflammation) are the most widely recognized subgroups (Ojanguren et al., 2025). Increasing evidence has indicated that dysbiosis of the human microbiota contributes to the development of these phenotypes, with specific microbial signatures associated with distinct immunological profiles.

In the Th2-high phenotype, characteristic alterations of the respiratory microbiota have been observed. Clinical studies demonstrated that patients with severe eosinophilic asthma exhibited significantly greater abundances of *Haemophilus* and *Streptococcus* in bronchial specimens compared with healthy controls or non-eosinophilic asthma patients, correlating positively with sputum eosinophil counts (Versi et al., 2024). Metagenomic sequencing of induced sputum from 55 asthma patients and 12 healthy individuals revealed that the α-diversity of the lower respiratory tract microbiota was significantly higher in Th2-high asthma compared to non-Th2 asthma, accompanied by a reduction in Proteobacteria abundance. Further analysis showed a denser microbial network structure in the Th2-high group, while the relative abundance of Actinobacteria correlated positively with eosinophil counts (Wang W. et al., 2024).

Notably, differences in microbial communities across asthma phenotypes extend beyond the respiratory tract to the gut. Metagenomic sequencing of fecal samples revealed that patients with eosinophilic asthma displayed a significant increase in gut fungal communities, particularly within the yeast phylum. In contrast, individuals with neutrophilic asthma exhibited increased levels of Bacillales (Yan et al., 2024). These findings suggest that both bacterial and fungal communities may serve as candidate biomarkers for distinguishing asthma phenotypes. Additional multi-cohort studies further supported these associations: reduced airway bacterial diversity and enrichment of Proteobacteria were linked to corticosteroid resistance and neutrophilic inflammation (Durack et al., 2020), whereas early-life depletion of gut microbial diversity increased the risk of persistent wheeze and subsequent asthma (Kim and Bunyavanich, 2025). Moreover, loss of butyrate-producing taxa such as *Faecalibacterium prausnitzii* correlated with impaired SCFA production and poor asthma control (Fujimura et al., 2016).

**Figure 2:**
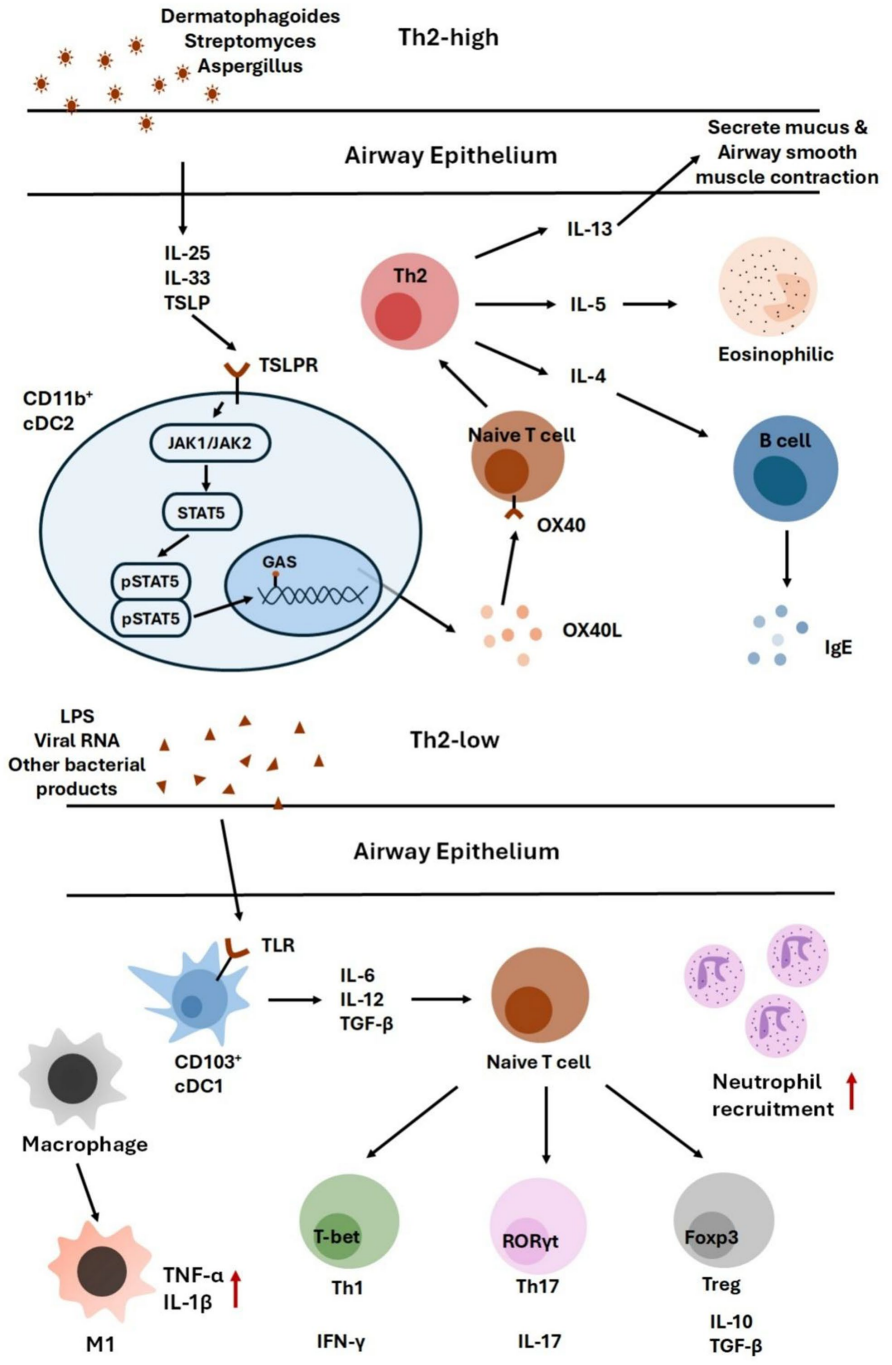
This figure summarizes how distinct microbial exposures contribute to different asthma endotypes. Th2-high/eosinophilic inflammation is driven by epithelial cytokines such as IL-25, IL-33, and TSLP, which amplify Th2 responses. In contrast, Th2-low/neutrophilic inflammation arises from TLR activation, leading to Th1/Th17-mediated immune responses.

To illustrate these links, Figure 2 summarizes how specific microbial exposures shape asthma endotypes. Th2-high/eosinophilic inflammation is primarily driven by epithelial cytokines (IL-25, IL-33, TSLP) that amplify Th2 responses, whereas Th2-low/neutrophilic inflammation is more closely associated with TLR-mediated activation of Th1/Th17 pathways. Collectively, these findings provide a mechanistic framework connecting microbial dysbiosis with the heterogeneity of asthma phenotypes.

### 3.2 Immunoregulatory functions of microbiota-derived metabolites

Microbial metabolites represented key mediators through which the gut microbiota influenced host immune regulation and contributed to asthma pathogenesis (Ge et al., 2025). Among these, short-chain fatty acids (SCFAs)—including acetate, propionate, and butyrate—were the most extensively studied. Produced in the intestinal lumen via dietary fiber fermentation, SCFAs entered systemic and lymphatic circulation and reached the lungs, where they modulated airway inflammation and immune balance.

Experimental studies demonstrated that butyrate bound to G protein–coupled receptor 43 (GPR43), inhibited the p38 MAPK/NF-κB signaling pathway, and suppressed IL-13 expression. This mechanism reduced Tfh13 cell–mediated production of high-affinity IgE and alleviated allergic airway responses (Yu et al., 2025). Similarly, propionate acted on GPR41 in dendritic cells, promoting the migration of intestinal Tregs to the lungs and limiting excessive Th2 inflammation (Mann et al., 2024). Beyond SCFAs, tryptophan-derived metabolites such as indole, serotonin, and indole-3-aldehyde were found to be enriched in low asthma-risk environments. Network analyses indicated that these protective metabolites were associated with cohabiting microorganisms such as Actinobacteria, suggesting that environmental microbial exposure could shape immune tolerance through metabolite production (Sun et al., 2022).

Not all microbial metabolites exerted protective effects. Animal studies revealed that 4-methylpentanoic acid, generated via branched-chain amino acid metabolism, was elevated in asthma models and positively correlated with IL-5 and IL-13 expression (Liu Y. H. et al., 2025). In addition, LPS released during gut dysbiosis could translocate into the bloodstream, activate TLR4 signaling in airway epithelial and stromal cells, and induce OSM expression. OSM drove mucus hypersecretion and promoted Th2/Th17 inflammation, leading to eosinophil and neutrophil infiltration (Headland et al., 2022; Zhang et al., 2025). However, previous investigations of LPS exposure and asthma yielded inconsistent outcomes, likely reflecting differences in experimental models, treatment timing, and dosage (Zakeri and Russo, 2018). Collectively, these findings suggested that LPS exerted a bidirectional influence on immune responses in asthma, with effects depending on exposure context.

In summary, microbial metabolites exerted diverse immunoregulatory effects in asthma, ranging from protective activities such as maintaining epithelial integrity and promoting Treg differentiation, to pathogenic actions including mucus hypersecretion, eosinophilic infiltration, and Th17-driven inflammation. These effects appeared to depend on metabolite type, concentration, and cellular targets, highlighting the complexity of metabolite-mediated immune modulation. Table 2 provides a comprehensive summary of the immunoregulatory mechanisms of major microbiota-derived metabolites in asthma pathogenesis.

**Table 2:** Immunoregulatory mechanisms of microbiota-derived metabolites in asthma.

Derivative	Source microbiota/origin	Study setting	Mechanism of action	Effect on immunity and asthma
Short-chain fatty acids				
Butyrate	*Roseburia*, *Faecalibacterium*	*In vivo*	Binds to GPR43 on Tfh13 cells, inhibits high-affinity IgE production; enhances Treg function (Yu et al., 2025)	Reduces airway eosinophil infiltration; promotes immune tolerance
Propanoic acid (propionate)	High-fiber diet microbiota	*In vivo*	Activates GPR41 on DCs, enhances bone marrow hematopoiesis, inhibits Th2 differentiation (Trompette et al., 2014)	Alleviates eosinophilic inflammation
Bile acids and derivatives				
3β-Hydroxydeoxycholic acid	*Bacteroides*	*In vivo*, *Vitro*	Binds to FXR on DCs, downregulates proinflammatory factors, induces FoxP3 + Treg differentiation (Campbell et al., 2020)	Promotes anti-inflammatory response and tolerance
3-Oxolithocholic acid	Actinobacteria, firmicutes	*In vivo*, *Vitro*	Inhibits RORγt transcription activity, suppressing IL-17a expression and Th17 differentiation (Yao, 2024; Hang et al., 2019)	Reduces neutrophil infiltration
Isoallolithocholic acid	*Bacteroides*	*In vivo*, *Vitro*	Enhances mitoROS production and H3K27ac in FoxP3 promoter, driving Treg differentiation (Yao, 2024; Hang et al., 2019)	Enhances Treg-mediated suppression of inflammation
Bile acids	*Bacteroides*	*In vivo*	Bind to nuclear receptors, stimulate IL-33 production and ILC2-mediated IL-5 secretion (Arifuzzaman et al., 2022)	Triggers eosinophilia and type 2 immune response
Tauroursodeoxycholic acid	–	*In vivo*	Binds to ATF6α, reduces allergen-induced UPR and serum IgE levels (Nakada et al., 2019)	Attenuates ER stress and allergic sensitization
Tryptophan metabolites				
Indole-3-propionic acid	*Clostridium*	*In vivo*	Maintains mitochondrial function in airway epithelium, reduces ROS, inhibits IL-6/TNF-α release (Perdijk et al., 2024)	Protects epithelial barrier; reduces inflammation
Kynurenine	–	*In vivo*	Binds AhR, induces epithelial CTH-mediated H₂S synthesis via IDO1-Kyn axis (Arias et al., 2023; Miao et al., 2024)	Increases Treg proportion, reduces Th17 infiltration, alleviates symptoms
Indole-3-carbaldehyde	*Bifidobacterium longum*	*In vivo*	Binds AhR, inhibits IL-17 and other inflammatory cytokines, increases Treg proportion (Zhang et al., 2020; Hufnagl et al., 2020; Wang H. et al., 2024)	Relieves inflammation
Others				
12,13-diHOME	–	*In vivo*	Alters PPARγ-regulated gene expression in DCs, reduces anti-inflammatory cytokine secretion and Treg numbers (Levan et al., 2019)	Destroys immune tolerance, promotes allergic inflammation
Lipopolysaccharide (LPS)	*Klebsiella pneumoniae*	*In vivo*	Binds TLR4, induces OSM expression, stimulating mucus hypersecretion and Th2/Th17 inflammation (Headland et al., 2022)	Promotes eosinophil and neutrophil infiltration into airways

AhR, Aryl hydrocarbon receptor; CTH, Cystathionine γ-lyase; DC, Dendritic cell; FXR, Farnesoid X receptor; GPR, G protein-coupled receptor; H3K27ac, Histone H3 lysine 27 acetylation; ILC2, Type 2 innate lymphoid cell; mitoROS, Mitochondrial reactive oxygen species; PPARγ, Peroxisome proliferator-activated receptor gamma; RORγt, Retinoic acid receptor-related orphan receptor gamma t; Treg, Regulatory T cell; UPR, Unfolded protein response.

### 3.3 Microbial biomarkers in asthma diagnostics and prognostication

Conventional prediction of acute asthma exacerbations had relied largely on clinical symptoms and lung function tests. However, these indicators often lag behind inflammatory progression, limiting their utility for early detection. Recent studies suggested that alterations in the airway and gut microbiota contributed to both asthma pathogenesis and acute exacerbations, with microbial composition changes emerging as sensitive predictive biomarkers. For example, elevated abundances of *Haemophilus influenzae* and *Moraxella* in sputum from patients with severe asthma correlate with increased sputum eosinophil counts and can predict exacerbation events (Heaney et al., 2016), Within the gut, *Ligilactobacillus murinus* (LGG) suppressed excessive inflammation through the TLR2/IL-10 pathway, and its fecal abundance was proposed as a marker of systemic immune status and a predictor of post-infection exacerbations (Hu et al., 2022). In addition to compositional markers, microbiota-derived metabolites provided functional insights: short-chain fatty acids (SCFAs) reduced airway eosinophil infiltration by binding to GPR41/43 and inhibiting histone deacetylase (HDAC), thereby reflecting the anti-inflammatory potential of the microbiota-host interaction. Furthermore, integrating microbial markers with established clinical indicators improved prognostic accuracy. A combined evaluation of eosinophil counts (EOS) and FeNO demonstrated greater predictive value for exacerbation events when both metrics were elevated, compared to either alone (Meulmeester et al., 2025). In summary, evidence indicated that the gut microbiota and its metabolites represented promising biomarkers for asthma diagnostics and prognostication. Their incorporation into clinical practice may enhance risk stratification and facilitate precision management of asthma.

## 4 Therapeutic role of microbiota in asthma treatment

Corticosteroids remain the first-line therapy for suppressing airway inflammation in asthma. However, a subset of patients exhibits suboptimal responses, and systemic administration is associated with adverse effects including hoarseness, oral candidiasis, growth suppression, and hypothalamic–pituitary–adrenal axis suppression (Voo et al., 2022). These limitations have prompted growing interest in complementary therapeutic strategies that target the gut–lung axis.

**Figure 3:**
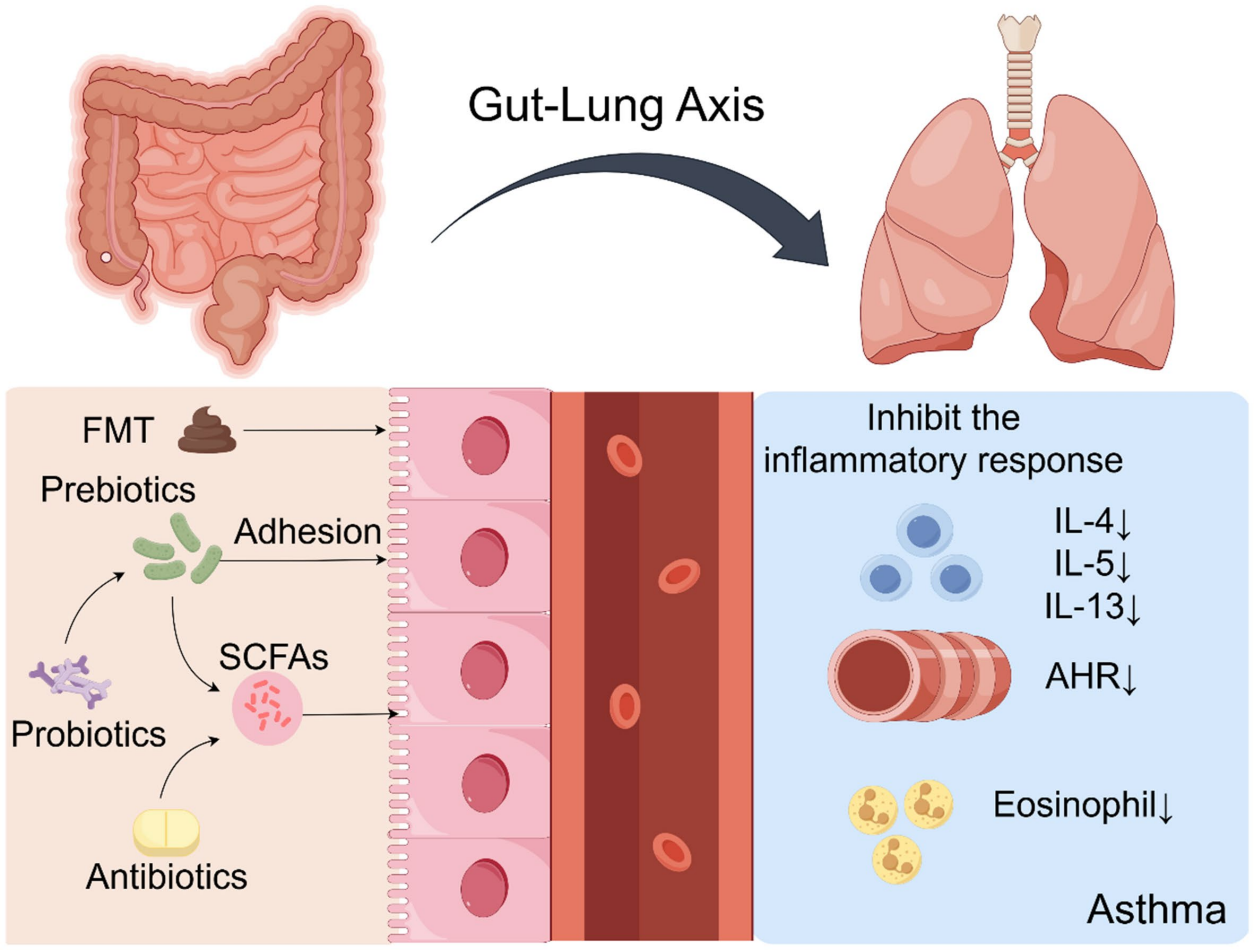
This schematic illustrates microbiota-targeted therapeutic approaches, including probiotics, prebiotics, and FMT. These interventions alleviate airway inflammation and symptoms by restoring microbial balance, enhancing SCFA production, and suppressing pro-inflammatory cytokines (e.g., IL-5, IL-13) and eosinophilic activity.

Accumulating evidence indicates that intestinal dysbiosis is a modifiable risk factor contributing to asthma pathogenesis (Liu Y. et al., 2025). Accordingly, microbiota-targeted interventions have emerged as promising approaches to restore immune homeostasis and improve clinical outcomes. These strategies include probiotics, prebiotics, synbiotics, fecal microbiota transplantation (FMT), precision antibiotic regimens, and targeted modulation of microbial metabolites. Their proposed mechanisms and available evidence are summarized in Table 3. Mechanistically, these interventions modulate disease processes by restoring microbial balance, increasing SCFA production, and downregulating type 2 inflammatory pathways, including the suppression of IL-5 and IL-13 as well as eosinophilic activity. Figure 3 illustrates the major pathways through which microbiota-based interventions may alleviate airway inflammation and clinical symptoms.

**Table 3:** Microbiota-targeted therapeutic strategies for asthma.

Intervention	Agent/object	Mechanism of action	Clinical/experimental effect
Probiotics	*Lactobacillus* spp.	Promote Th1/Th2 balance; reduce IgE and Th2 cytokines (Liu et al., 2016)	Improve Asthma Control Test (ACT) scores and pulmonary function (Chen et al., 2010; Li et al., 2022)
		Produce SCFAs to regulate DCs, macrophages, and T cells (Leser and Baker, 2024)	Reduce asthma exacerbation frequency and severity (Drago et al., 2022)
		Enhance Treg activity; suppress IL-6 and IL-17 (Garcia-Castillo et al., 2020)	Prevent house dust mite (HDM)-induced asthma (Spacova et al., 2020)
	*Bifidobacterium* spp.	Stimulate Treg differentiation via IL-10 and Foxp3 (Perdijk et al., 2024)	Decrease childhood asthma incidence (Berni Canani et al., 2017)
Prebiotics	GOS/FOS	Ferment to SCFAs, inhibiting DC-mediated Th2 priming (Verstegen et al., 2021)	Reduce AHR and levels of TNF-α/CCL17 (Verstegen et al., 2021)
	Inulin	Restructure gut microbiota composition (Hassanzad et al., 2019)	Improve control of airway inflammation (Hassanzad et al., 2019)
	HMO	Generate immunomodulatory acetate via *Bifidobacterium* (Slater et al., 2024)	Promote infant immune maturation (Slater et al., 2024)
Synbiotics	Probiotic + Prebiotic comb.	Increase airway Treg populations (Hassanzad et al., 2019)	Reduce recurrent wheezing and medication use (Meirlaen et al., 2021)
FMT	Healthy donor microbiota	Transfer complete microbial consortia (Kang and Cai, 2018)	Reshape pulmonary immune cell profiles (Kang and Cai, 2018)
	Asthma patient microbiota	Enrich *Bacteroides fragilis* (Park et al., 2020)	Induce Th17 airway inflammation (Park et al., 2020)
Metabolites	SCFAs (e.g., Butyrate)	Activate GPR41/43; inhibit HDAC (Hassanzad et al., 2019; Meirlaen et al., 2021)	Promote eosinophil apoptosis; suppress mucus hypersecretion (Meirlaen et al., 2021)
		Drive fetal lung Treg development via placental transfer (Yang and Cong, 2021)	Prevent offspring asthma (Yang and Cong, 2021)
	High-fiber diet	Boost butyrate-producing *Clostridiales* (Slater et al., 2024)	Attenuate allergic lung inflammation (Slater et al., 2024)
Antibiotics	Azithromycin (AZM)	Reduce sputum TNF/TNFR2 in T2-low asthma (Huang et al., 2025)	Decrease airway inflammation biomarkers (Huang et al., 2025)
		Activate sphingomyelin metabolism (Huang et al., 2025)	Inhibit goblet cell hyperplasia and IL-5/IL-13 (Huang et al., 2025)
		Increase *Clostridiales* abundance (Park et al., 2020)	Suppress Th2 inflammation (Park et al., 2020)
		Reshape airway microbiota in HDM models (Park et al., 2020)	

AHR, Airway hyperresponsiveness; DC, Dendritic cell; FMT, Fecal microbiota transplantation; FOS, Fructooligosaccharides; GOS, Galactooligosaccharides; HDAC, Histone deacetylase; HDM, House dust mite; HMO, Human milk oligosaccharides; SCFA, Short-chain fatty acid; Treg, Regulatory T cell.

### 4.1 Probiotics

Probiotics, defined as “live microorganisms” that confer health benefits when administered in adequate amounts (Peng et al., 2024). Emerging evidence from both preclinical and clinical studies demonstrates that probiotic supplementation exerts beneficial immunomodulatory effects in asthma, acting primarily through the gut–lung axis as a primary upstream driver of host–microbe interactions (Tashiro et al., 2025). As summarized in Figure 3, probiotic interventions restore gut microbial homeostasis, promote anti-inflammatory metabolite production, and modulate systemic immune signaling, thereby influencing pulmonary immunity.

Mechanistically, probiotic-derived signals are first sensed by gut mucosal immune cells via PRRs, leading to the activation of innate immune pathways and subsequent trafficking of immune cells to the lungs. For example, oral probiotic supplementation enhances intestinal Th1 responses and facilitates the migration of CD4^+^ IFN-γ^+^ T cells to the airways, rebalancing the Th1/Th2 axis and attenuating allergen-induced sensitization. This process is accompanied by reduced serum levels of IgE and Th2-associated cytokines, including IL-5, IL-6, IL-8, and IL-17 (Garcia-Castillo et al., 2020). In parallel, probiotic-derived SCFAs act as critical intermediate modulators by regulating the activity of DCs, macrophages, and T cells, enhancing Treg differentiation and suppressing pro-inflammatory cytokine production (Leser and Baker, 2024). These interconnected pathways illustrate how probiotics influence asthma pathogenesis through a hierarchical mechanism: gut microbial modulation as the upstream driver, epithelial and metabolic signaling as intermediate modulators, and immune cell reprogramming as downstream effectors.

The therapeutic potential of probiotics in asthma has been investigated through a combination of preclinical studies and randomized controlled trials (RCTs), although the strength of evidence remains heterogeneous (Drago et al., 2022; Li et al., 2020). Preclinical models consistently demonstrate that oral or inhaled administration of *Lactobacillus* strains can reduce allergen-induced airway inflammation, suppress AHR, and modulate Th2-driven immune responses (Spacova et al., 2020). These findings provide essential mechanistic insights but require careful translation to human disease contexts.

In clinical settings, several RCTs have evaluated the efficacy of probiotics as adjunctive interventions in asthma management, with mixed but generally favorable outcomes. In a double-blind RCT, Li et al. reported that 8-week supplementation with *Lactobacillus reuteri* CCFM1040 significantly improved respiratory symptoms in patients with allergic rhinitis and asthma, as reflected by higher Asthma Control Test (ACT) scores compared to placebo (Li et al., 2022). Two additional RCTs in pediatric populations demonstrated that daily intake of Lactobacillus strains reduced FeNO levels and improved lung function parameters, suggesting attenuation of airway inflammation (Chen et al., 2010). Beyond symptom control, probiotic supplementation has shown promise in preventing asthma development. An RCT involving 422 children found that administration of *Lactobacillus salivarius* DSM 22775 significantly reduced the frequency and severity of asthma exacerbations (Drago et al., 2022). Moreover, a three-year longitudinal RCT demonstrated that *Lactobacillus rhamnosus GG* (LGG)-fortified formula lowered asthma incidence among children with cow’s milk allergy (Berni Canani et al., 2017). These clinical findings collectively support a moderate but growing evidence base for probiotic use in asthma, while also underscoring the need for larger, well-controlled trials to optimize strain selection, dosage, and intervention timing.

In addition to their standalone effects, probiotics can act as adjuvant therapies that enhance the efficacy of conventional asthma treatments. Preclinical studies have shown that co-administration of *Bifidobacterium* and LGG with glucocorticoids exerts synergistic anti-inflammatory effects, leading to greater suppression of AHR and airway inflammation than glucocorticoid monotherapy (Voo et al., 2022). These findings suggest that probiotic co-therapy may permit lower glucocorticoid dosages while maintaining clinical efficacy, potentially minimizing treatment-related adverse effects.

Probiotics have also been investigated as adjuncts to allergen-specific immunotherapy (AIT). Clinical studies indicate that the combination of probiotics with AIT significantly improves asthma symptom control compared to AIT alone (Liu et al., 2016). Mechanistically, this effect involves enhanced induction of antigen-specific regulatory B10 cells and reduced serum levels of allergen-specific IgE, thereby augmenting immune tolerance. Given the emerging role of the lung microbiota in asthma pathogenesis, inhalation-based probiotic delivery has been proposed as a novel strategy to directly modulate the airway microbial community. By targeting the respiratory tract, this approach may enhance local immune regulation and reduce inflammation, complementing the systemic effects of oral supplementation. Although clinical data remain limited (Glieca et al., 2024; Nicola et al., 2024), early findings suggest that inhaled probiotics could represent a promising avenue for targeted microbiota modulation in asthma management.

Collectively, current evidence supports the therapeutic potential of probiotics as both preventive and adjunctive interventions in asthma management. Preclinical studies consistently demonstrate mechanistic efficacy through modulation of the gut–lung axis, while clinical trials provide emerging, though heterogeneous, support for improvements in airway inflammation and symptom control. Importantly, probiotics are generally well tolerated and carry a favorable safety profile, making them attractive candidates for long-term disease modulation. Nevertheless, the clinical evidence base remains limited by small sample sizes, short follow-up durations, and substantial heterogeneity in probiotic strains, dosages, and patient populations. To advance probiotic therapy toward clinical translation, future research should prioritize large, well-designed randomized controlled trials to determine optimal microbial strains and combinations, standardized dosing regimens, and critical intervention windows (e.g., perinatal versus postnatal periods). Integrating microbiome profiling and immunophenotyping into clinical trial design will be essential for identifying patient subgroups most likely to benefit and for elucidating causal mechanisms. Overall, while probiotics represent a promising and biologically plausible therapeutic avenue, their incorporation into routine asthma care will require more rigorous evidence to define efficacy, durability of effects, and patient-specific therapeutic strategies.

### 4.2 Prebiotics and synbiotics

Prebiotics are defined as indigestible organic substrates that selectively stimulate the growth and metabolic activity of beneficial commensal microorganisms, thereby contributing to host health by restoring microbial homeostasis (Fan et al., 2025). In the context of asthma prevention and management, commonly studied prebiotics—including inulin, fructooligosaccharides (FOS), and galactooligosaccharides (GOS)—are fermented by the gut microbiota to produce SCFAs. SCFAs act as key intermediate immunomodulators, inhibiting DC–mediated Th2 immune priming and attenuating allergic airway inflammation (Blanco-Pérez et al., 2021). Beyond these synthetic substrates, human milk oligosaccharides (HMOs)—major components of breast milk—play a pivotal role in shaping the neonatal gut microbiota. Metabolism of HMOs by *Bifidobacterium* species generates high concentrations of acetate, which are critical for early-life immune programming (Slater et al., 2024). Clinically, synbiotic formulations combining prebiotics and probiotics have been shown to enhance gut microbial diversity and modulate immune responses in high-risk infants. RCTs indicate that such supplementation significantly reduces the incidence of wheezing episodes, suggesting potential for early-life asthma prevention (Hassanzad et al., 2019).

The therapeutic potential of prebiotic supplementation in asthma is increasingly supported by both mechanistic and clinical evidence. RCTs have shown that administration of GOS and inulin significantly reduces AHR and inflammatory biomarkers such as TNF-α and C-C motif CCL17, while concurrently improving lung function in patients with stable asthma (Verstegen et al., 2021). Inulin supplementation has been further associated with gut microbiota remodeling and enhanced disease control (Hassanzad et al., 2019). These clinical effects are closely linked to SCFA-mediated immune regulation, which promotes anti-inflammatory responses and fosters colonization of beneficial microbial taxa.

Experimental studies provide complementary mechanistic insights: mice maintained on high-fiber diets exhibit increased SCFA production and display significantly attenuated airway inflammation in experimental asthma models, largely through enhanced Treg responses (Slater et al., 2024). Evidence for synbiotics remains more limited, but early clinical trials indicate promising preventive effects. In a 12-week multicenter RCT involving 90 infants with atopic dermatitis, 7-month synbiotic supplementation reduced the incidence of recurrent wheezing and respiratory symptoms, along with decreased asthma medication use, although no significant differences in total IgE levels were observed (Meirlaen et al., 2021).

Collectively, while current findings highlight the mechanistic plausibility and early clinical benefits of prebiotics and synbiotics in asthma, the evidence base remains fragmentary and heterogeneous. Future research should focus on large-scale, longitudinal RCTs to define optimal substrate types, dosages, and intervention windows, and to identify patient subgroups most likely to benefit.

### 4.3 Fecal microbiota transplantation

Fecal microbiota transplantation (FMT) involves the transfer of functional microbial communities from healthy donors to recipients with the aim of restoring intestinal microbial homeostasis (Kang and Cai, 2018). Unlike probiotics, which typically introduce single or limited strains, FMT delivers a complex and stable microbial consortium, thereby enabling more durable engraftment and niche restoration. Through these mechanisms, FMT has shown therapeutic potential across several extraintestinal conditions—including metabolic, neuropsychiatric, autoimmune, and allergic disorders—by re-establishing colonization resistance and modulating immune responses. Within the framework of the gut–lung axis, FMT is hypothesized to act as an upstream microbial intervention capable of influencing pulmonary immunity via metabolic and immunological signaling.

Although no clinical trials have yet evaluated FMT specifically for asthma, accumulating preclinical evidence supports its immunomodulatory capacity. In murine models, transplantation of fecal microbiota from patients with asthma—characterized by enrichment of *Bacteroides fragilis*—into germ-free recipients induced T helper 17 (Th17)–polarized airway inflammation (Park et al., 2020). In antibiotic-treated respiratory disease models, FMT has been shown to reshape pulmonary immune cell landscapes, highlighting the bidirectional communication between gut and lung microbial communities. Compared with probiotic interventions, FMT provides superior microbial biomass and engraftment stability, potentially enabling more profound reconfiguration of host–microbiota interactions.

Despite its promise, the clinical translation of FMT for asthma faces several technical, economic, and safety challenges. Standardized fecal processing protocols and targeted delivery systems remain underdeveloped, and the procedure is currently 3–5 times more expensive per capita than conventional therapies (Hou et al., 2022). Safety concerns—including the risk of pathogen transmission and uncertain outcomes in immunocompromised or pregnant individuals—must be carefully addressed before clinical implementation. Future research should prioritize the development of selective microbial transplantation strategies, ideally supported by large-scale, well-controlled clinical trials. Establishing donor–recipient matching frameworks and optimizing microbial consortia composition will be essential for achieving precise immune modulation in asthma. Furthermore, integration of multi-omics approaches and immune phenotyping may enable better prediction of FMT outcomes and identification of responsive patient subgroups.

### 4.4 Targeting microbial metabolites

Microbial metabolites serve as critical intermediates linking intestinal microbial composition to systemic and pulmonary immune regulation. Among these, SCFAs—such as acetate, propionate, and butyrate—have been most extensively studied. In animal models, dietary fiber supplementation significantly increases SCFA production, which in turn suppresses allergic airway inflammation by promoting the differentiation and function of Tregs (Yang and Cong, 2021). SCFA-mediated induction of Tregs provides early protection against Th2-driven allergic phenotypes through microbiota-dependent immune education following intestinal colonization.

Early-life exposure represents a critical immunological window for SCFA-mediated effects. Experimental studies have demonstrated that microbial exposure after weaning fails to reverse asthma-related immune and phenotypic abnormalities, underscoring the importance of early colonization for immune programming. Notably, acetate can cross the placental barrier and promote fetal lung Treg development, thereby attenuating postnatal asthma susceptibility (Lee-Sarwar et al., 2020). These findings highlight the potential of maternal diet modulation and early-life microbial interventions as preventive strategies for asthma.

Beyond SCFAs, other microbial metabolite classes—including polyamines, tryptophan derivatives, and bile acids—play emerging roles in modulating immune homeostasis. Polyamines regulate epithelial integrity and inflammatory gene expression, while tryptophan metabolites act via the aryl hydrocarbon receptor to shape innate lymphoid and T cell responses. Secondary bile acids, such as TUDCA and β-muricholic acid, have been shown to suppress Th2 cytokine production and IgE levels, thereby attenuating allergic airway inflammation (Nakada et al., 2019). Although these pathways are less extensively characterized than SCFAs, they provide complementary mechanisms through which the microbiota can influence asthma pathogenesis.

Looking forward, future research should prioritize integrated metabolomics–immunology approaches to define the temporal dynamics and causal relevance of these metabolite classes in asthma. Clinical translation will require well-controlled dietary and microbial interventions aimed at selectively modulating metabolite production during critical developmental periods. Such strategies may ultimately enable precise, metabolite-targeted modulation of the gut–lung axis for asthma prevention and therapy.

### 4.5 Precision antibiotic therapy

Antibiotic-induced alterations of the gut microbiota exert profound effects on host immune regulation, particularly through disruption of the gut–lung axis. While broad-spectrum antibiotics are effective in controlling infections, their nonspecific antimicrobial activity perturbs intestinal microbial homeostasis, leading to decreased production of SCFAs and impaired Treg function (Donald and Finlay, 2024). In murine models, early-life vancomycin exposure selectively depletes butyrate-producing Clostridium populations, resulting in exacerbated allergic airway inflammation (Chen et al., 2022).

Among available antimicrobials, azithromycin (AZM) displays distinctive dual immunomodulatory properties. Clinical investigations have shown that prolonged AZM therapy significantly reduces sputum TNF/TNFR2 concentrations in non-eosinophilic, Th2-low asthma endotypes. Preclinical studies further demonstrate that AZM mitigates FMT–induced allergic airway inflammation by reducing BALF IL-5 levels and AHR, while concurrently remodeling airway microbial communities in HDM–challenged asthma models (Park et al., 2020). Mechanistically, AZM activates the sphingomyelin (SM) metabolic pathway, leading to reduced goblet cell proliferation and inhibition of Th2 cytokines, including IL-5 and IL-13, thereby attenuating type 2 airway inflammation (Huang et al., 2025). Experimental studies further reveal that antibiotic-naïve HDM-sensitized mice spontaneously develop heightened AHR and elevated Th2 cytokines (IL-4, IL-5, IL-13) accompanied by pronounced eosinophilic infiltration in BALF. 16S rRNA sequencing demonstrates that this inflammatory state is associated with significant gut dysbiosis, despite minimal changes in airway microbiota composition (Park et al., 2020). Collectively, these data underscore the bidirectional interactions between gut microbial communities and pulmonary immune responses and highlight the potential of AZM as both an antimicrobial and immunomodulatory agent.

Future research should aim to define precision antibiotic strategies that minimize collateral microbiota disruption while harnessing the immunomodulatory benefits of specific agents such as AZM. Integrating microbiome profiling with clinical phenotyping may enable individualized antimicrobial interventions that align with microbiota-targeted therapies for allergic asthma.

## 5 Summary and future perspectives

Microbial dysbiosis disrupts host homeostasis and plays a pivotal role in the pathogenesis of immune-mediated diseases, including asthma. As a multifactorial disorder shaped by both genetic and environmental influences, asthma pathogenesis, clinical phenotypes, and disease severity are closely linked to dynamic fluctuations in microbial communities within the respiratory and gastrointestinal systems. Although the pulmonary microbiota has a relatively low biomass, it engages in continuous crosstalk with the intestinal microbiota through lymphatic circulation, hematogenous dissemination, and micro-aspiration, establishing the bidirectional gut–lung axis. Through this axis, distal microbial communities exert context-dependent regulatory effects on pulmonary immune responses, thereby contributing to the inflammatory and heterogeneous nature of asthma.

Future research should prioritize several interrelated areas to advance mechanistic understanding and clinical translation. First, delineating phenotype–microbiota correlations represents a critical step toward precision medicine in asthma. Longitudinal cohort studies with larger sample sizes are needed to clarify how microbial composition and function relate to inflammatory phenotypes and disease severity in both pediatric and adult populations. Second, establishing causal links between microbial metabolites and asthma pathophysiology remains a key challenge. Integrating single-cell multi-omics with targeted metabolomics will enable high-resolution mapping of host–microbe interactions, identify functional microbial signatures, and uncover novel therapeutic targets. Third, standardizing interventional strategies and clinical trial protocols is essential for translating microbiota research into therapeutic applications. Microbial consortia engineering and rationally designed probiotics offer promising approaches to stably modulate host immunity, but their efficacy must be validated in well-controlled randomized clinical trials. Finally, future efforts should aim to integrate inter-kingdom microbial dynamics—including bacterial, viral, and fungal interactions—into holistic models of asthma pathogenesis. Such integrative frameworks will be crucial for developing individualized microbiota-targeted therapies that account for disease heterogeneity across phenotypes and endotypes.

Collectively, addressing these research priorities will deepen our understanding of the gut–lung axis in asthma and accelerate the development of mechanism-based, microbiota-informed strategies for disease prevention and treatment.

## Glossary

AHR: Airway hyperresponsiveness
Th: T helper cell
PRRs: Pattern recognition receptors
PAMPs: Pathogen-associated molecular patterns
DAMPs: Damage-associated molecular patterns
TLRs: Toll-like receptors
Treg: Regulatory T cell
ILC2s: Group 2 innate lymphoid cells
SCFAs: Short-chain fatty acids
IgA: Immunoglobulin A
IgE: Immunoglobulin E
IL: Interleukin
TSLP: Thymic stromal lymphopoietin
GMT: Gut microbiota transplantation
OSM: Oncostatin M
TUDCA: Tauroursodeoxycholic acid
TNF-α: Tumor necrosis factor-alpha
IFN-γ: Interferon-gamma
HDM: House dust mite
BALF: Bronchoalveolar lavage fluid
ACT: Asthma control test
AZM: Azithromycin
DC: Dendritic cell
FeNO: Fractional exhaled nitric oxide
FMT: Fecal microbiota transplantation
FOS: Fructooligosaccharides
GOS: Galactooligosaccharides
HDAC: Histone deacetylase
HMO: Human milk oligosaccharides
LPS: Lipopolysaccharide
Chrm4: Muscarinic receptor M4
NETs: Neutrophil extracellular traps
RCT: Randomized controlled trial
EOS: Eosinophil counts
LGG: *Ligilactobacillus murinus*
AIT: Allergen-specific immunotherapy
